# Review: Diagnostic Potential for Collaborative Pharyngitis Biomarkers

**DOI:** 10.1093/infdis/jiae416

**Published:** 2024-10-23

**Authors:** Nathan A Ledeboer, Jane M Caldwell, Bobby L Boyanton

**Affiliations:** Department of Pathology and Laboratory Medicine, Medical College of Wisconsin, Milwaukee; Medavera, Inc, Springfield, Missouri; Department of Pathology and Laboratory Medicine, Arkansas Children's Hospital, Little Rock, AR, USA; Department of Pathology and Laboratory Medicine, University of Arkansas for Medical Sciences, Little Rock, Arkansas, USA

**Keywords:** pharyngitis, biomarkers, nucleic acid amplification tests, antibiotic stewardship, rapid antigen testing

## Abstract

Pharyngitis is an inflammatory condition of the pharynx and/or tonsils commonly seen in both children and adults. Viruses and bacteria represent the most common encountered etiologic agents—yeast/fungi and parasites are infrequently implicated. Some of these are predominantly observed in unique populations (eg, immunocompromised or unvaccinated individuals). This manuscript (part 3 of 3) summarizes the current state of biomarker diagnostic testing and highlights the expanding role they will likely play in the expedited diagnosis and management of patients with acute pharyngitis. Biomarkers, in conjunction with rapid antigen and/or nucleic acid amplification testing, will likely become the standard of care to accurately diagnose the etiologic agent(s) of pharyngitis. This novel testing paradigm has the potential to guide appropriate patient management and antibiotic stewardship by accurately determining if the cause of pharyngitis is due to a viral or bacterial etiology.

## KNOWLEDGE GAPS IN DIAGNOSTICS

Bacteria and viruses are the most common etiologic agents of pharyngitis. Clinical history and observed physical signs/symptoms cannot accurately determine the infectious agent, even when employed by experienced healthcare providers (HCPs) [1]. If this dichotomy was not sufficiently problematic, additional factors (coinfections, asymptomatic colonization, and subclinical infection) further impede the ability of the HCP to accurately diagnose and treat the patient. Using a combinatorial approach, HCPs integrate clinical scoring systems with laboratory data, including rapid antigen, culture, biomarkers, and/or nucleic acid amplification tests (NAATs) to facilitate the correct diagnosis. These biomarkers and NAATs have advanced the diagnostic process by rapidly identifying specific pathogens with high analytical specificity and sensitivity. In addition, multiplex NAATs are being developed; these simultaneously detect both common and less frequently encountered pathogens. However, rapid antigen, culture, and NAATs all share similar disadvantages, including the inability to distinguish between asymptomatic colonization, subclinical infection, coinfections, and, when excluding culture, viable versus nonviable organisms. New paradigms are needed to facilitate rapid and accurate diagnosis and treatment when needed.

## A NEW DIAGNOSTIC PARADIGM

For more than a century, infectious diseases diagnostics relied upon detection of an offending pathogen from the site of infection without considering the role of the host. The ability to evaluate the host response is a relatively new approach to identify and monitor infectious diseases using an array of biomarkers to enhance HCPs’ diagnostic acumen. Unlike pathogen detection modalities that only identify the presence of a single or multiple pathogen(s), host response diagnostics assess an individual’s immune response to the responsible pathogen(s).

The host response to infection involves a complex interplay between innate and adaptive immunity. When the immune system initially encounters a pathogen, the first line of defense is the fast-acting “nonspecific” innate immune response [2]. This process relies on the host's ability to rapidly recognize pathogens, typically via Toll-like receptors, and subsequently activate the complement cascade [2]. Complement activation is followed by disruption of the microorganism cell membrane and pathogen targeting for phagocytosis [2]. The innate response is followed by the slow-acting “specific” adaptive immune response, which involves a complex interplay between both T- and B-lymphocytes [2]. The end game is the development of specific antibodies that target the offending pathogen(s), and each step of these complex processes provides an opportunity for the development of host response diagnostic testing solutions [2].

Host biomarkers including white blood cell (WBC) counts as general indicators of inflammation and blood levels of C-reactive protein (CRP) and procalcitonin (PCT) are widely studied and used. Some newer potential point-of-care (POC) or near-care laboratory technologies including flow cytometry [3, 4], lateral flow devices [5], and injected nanoparticle sensors [6] may expand the host response diagnostics that can be used to distinguish active infection quickly and accurately from colonization and other conditions. A complex diagnosis may require a complex testing strategy that integrates artificial intelligence (AI)–generated models and algorithms, clinical scores, and host-derived biomarkers, plus quantitative bacterial/viral nucleic acid amplification (NAA) detection methods. In this review, we discuss several promising host response assays that may play a role in pharyngitis diagnosis.

## PROTEIN BIOMARKER EVALUATIONS

There are several potential protein biomarkers associated with the immune response to infection that may be leveraged in POC diagnostics. CRP and PCT are 2 of the best-known biomarkers used in routine care [7]. CRP is a nonspecific acute-phase protein produced in response to inflammatory cytokines [5]. PCT is a small hormone (32 amino acids) produced by the thyroid C-cells that has a positive correlation with bacterial infection and sepsis but not viral infections [8]. PCT has been reported to be a sensitive biomarker of severe bacterial infection as it is specifically released in response to bacterial toxins [9–11]. One study evaluated a small cohort of children with bacterial tonsillopharyngitis, nonbacterial tonsillopharyngitis, and healthy controls (n = 15 for each category; 45 children total) [12]. They found median PCT concentrations in blood samples in the bacterial group to be significantly higher (0.374 ng/mL) as compared to the nonbacterial (0.105 ng/mL) and control (0.03 ng/mL) groups. In the same study, serum concentrations of CRP were significantly higher in the bacterial and nonbacterial groups than the control group. The median (range) values of CRP were 50 (22.4–71.1), 23.6 (5.9–61.2), and 2.6 (0.9–6) mg/L in the bacterial, nonbacterial, and control groups, respectively (*P* < .01). Data on PCT, CRP, erythrocyte sedimentation rate (ESR [Westergren method]), and leukocyte counts were statistically analyzed using the nonparametric Mann-Whitney *U* test. Sensitivity and specificity of each parameter were derived from the receiver operating characteristic (ROC) curve and the Youden index was calculated to determine optimal cutoff values. The best cutoff value for PCT to distinguish between bacterial and nonbacterial tonsillopharyngitis was 0.2275 ng/mL with 73% and 87% sensitivity and specificity, respectively. The best distinguishing cutoff value for CRP was 39.2 mg/L with 80% and 73% sensitivity and specificity, respectively. Neither ESR or leukocyte count exhibited high specificity in differentiating between bacterial and nonbacterial infections. When using ROC curves, the authors concluded that PCT had a superior specificity for detection of bacterial tonsillopharyngitis when compared with CRP or leukocytes [12].

A more recent study by Yu et al enrolled 18 children with pharyngitis (Centor scores of 4 or 5) and 21 noninfected controls to evaluate host response with WBC, CRP, PCT, and sequencing of RNA from peripheral blood leukocytes [13]. WBC, CRP, and PCT were higher in those patients with pharyngitis compared to asymptomatic controls, including those carrying group A *Streptococcus* (GAS) asymptomatically. Transcriptional profiles of children with symptomatic GAS were distinct from all other groups. They found that expression of either of 2 genes, *CD177* and *TLR5*, could accurately distinguish between symptomatic and asymptomatic GAS. *CD177* produces a cell surface glycoprotein involved in neutrophil activation and transmigration, and *TLR5* produces a Toll-like cell surface receptor that stimulates proinflammatory pathways [13]. TLR5's use as a biomarker was surprising since it was known to bind bacterial flagellin, yet *Streptococcus pyogenes* (GAS) bacteria do not have these structures [13]. The authors concluded that a toolbox approach using both traditional measures of inflammation and molecular markers of host gene expression could adequately distinguish between symptomatic and asymptomatic GAS. Implementing the testing protocol described by Yu et al [13] with contemporary technology necessitates substantial adaptation of clinical workflows, given that the majority of patients presenting with pharyngitis at primary care or urgent care settings do not typically undergo blood draws or necessitate extensive follow-up. The advancement of Clinical Laboratory Improvement Amendments (CLIA)–waived technologies capable of conducting tests at or near the POC, without the need for venipuncture, holds promise for enhancing the clinical applicability of Yu and colleagues' testing approach.

Studies investigating acute respiratory infections (ARIs) may have implications for pharyngitis diagnoses. In a recent systematic review of 13 studies conducted in Europe, Russia, and Asia, Smedemark et al investigated the use of CRP point-of-care tests (POCTs) for ARIs in the primary care setting [14]. Twelve trials (10 218 total participants, 2335 of which were children) evaluated a CRP POCT, and 1 trial (317 adults) evaluated a PCT POCT. In their meta-analysis, the authors conclude that CRP POCT rapid results (2–3 minutes) likely reduced the number of individuals given an antibiotic prescription at the initial consultation. CRP POCT was unlikely to affect patient safety in regard to clinical recovery within a 7- or 28-day follow-up or to increase hospital admissions. Mortality within 28 days was also unlikely to occur. The authors cautioned that clinicians should be aware of different algorithms and cutoff points when using CRP tests. A CRP level <20 mg/L has been suggested as a cutoff for antibiotic use, but is not validated in the literature for children and older adults with comorbidities [14].

To date, only 1 study has investigated the role of rapid (20 minutes) PCT POCT in individuals with ARI in the primary care setting [15]. Compared with usual care, PCT POCT led to a 26% reduction in the probability of 28-day antibiotic prescription without affecting patient safety [15]. Therefore, in the previously mentioned meta-analysis, the authors felt they could not comment on PCT POCT's impact on antibiotic use or other primary and secondary patient outcomes due to a paucity of data [14]. They recommended further studies to evaluate PCT and other potential new biomarkers as POCTs in primary care and also stated that CRP was the only biomarker available for POC in primary care having data, which supported its use in guiding antibiotic prescriptions for ARIs [14]. According to the US Food and Drug Administration (FDA) website, there are no CRP or PCT biomarker tests for use at POC with CLIA-waived status [16]; however, European countries are already incorporating POCTs in clinical diagnostic settings. Meta-analyses from Denmark and other non-US countries [14, 17, 18] have quantified the reduction of unnecessary antibiotic use and improved patient outcomes when integrating POC biomarkers in primary care.

## NOVEL IMMUNE BIOMARKERS

Myxovirus resistance protein (MxA) is an antiviral protein that is expressed by multiple cell types (including mononuclear cells, plasmacytoid dendritic cells, and myeloid cells) during viral infection [19]. In an exploratory study, the diagnostic value of monocyte MxA expression was evaluated in adults with viral, bacterial, or coinfections [3]. Monocyte MxA was measured via flow cytometry from a cohort of 61 adults with viral, bacterial, and coinfections. Patients receiving immunosuppressive therapy were also included. Results indicated that monocyte MxA expression was significantly higher in virus-infected versus bacteria-infected individuals (83.3 vs 33.8 mean fluorescence intensity [MFI]; *P* < .0001), but not in individuals with viral–bacterial coinfections (53.1 MFI). Importantly, monocyte MxA expression in patients with viral infections was not affected by immunosuppressive therapies, indicating that MxA may also be a useful biomarker in immunocompromised patients [3].

Another study evaluated MxA and CRP simultaneously when taken from finger-stick blood using a rapid, single-use, POC lateral flow device [5]. Increased CRP levels were indicative of bacterial infection while increased MxA levels were indicative of viral infection without regard to CRP levels. Among 520 individuals with ARIs in the outpatient setting, the lateral flow test demonstrated high sensitivity (93.2%) and negative predictive value (NPV) (98.7%) with moderate specificity (88.4%) and positive predictive value (PPV) (58.1%) for classifying bacterial infections. Viral infections were classified with moderate sensitivity (70.3%), specificity (88.0%), PPV (89.7%), and NPV (66.7%). Because results were available within 10 minutes, this rapid POCT may serve as a screening test that helps reduce the likelihood of bacterial infection from consideration, but further studies are needed in defined clinical scenarios (eg, acute pharyngitis, pneumonia).

No single biomarker, but rather combinations thereof may be needed to demonstrate potential clinical utility [20]. For example, an FDA-cleared host response test, MeMed BV [21], is a rapid assay designed to distinguish between bacterial and viral infections with the potential reduction of diagnostic uncertainty at POC [22]. This test uses an algorithm combining 3 proteins—TRAIL (a member of the tumor necrosis family), IP-10 (interferon-γ–induced protein-10), and CRP—to capture the host's immune response to infection and points to the source using multiple biological pathways [22]. TRAIL plays a role in regulating programmed cell death, a major response to viral infections [22]. IP-10 is a small cytokine involved in chemotaxis and cell growth inhibition; it is induced in both bacterial and viral infections but is more highly expressed in response to viral infections. CRP, as mentioned previously, is an inflammatory biomarker that responds to bacterial infection [22]. Multiple clinical studies have validated BV’s accuracy [22–29] and there are several studies citing the sensitivity and specificity of BV in various settings such as emergency departments and urgent care facilities. Although none of the reports focus on pharyngitis alone but examine both children and adults presenting with diverse clinical syndromes, the data do not exclude pharyngitis and the results point to BV’s potential utility in diagnosing bacterial pharyngitis.

BV was tested in a real-world setting with regard to its impact on antibiotic stewardship in urgent care [22]. The use of antibiotics before and after implementation of BV testing in 3 urgent care centers (152 patients) was analyzed. In total, 86.2% had either a bacterial or viral BV result. The authors reported that the BV result impacted HCP decisions in 87% of cases (*P* < .05). The authors concluded that BV impacted patient management in the urgent care setting by supporting appropriate antibiotic use [22].

Another real-world study analyzed a retrospective cohort (n = 60) using plasma samples from pediatric patients with acute febrile illness who visited the emergency department of 1 hospital [30]. The BV results showed excellent agreement with the manufacturer's laboratory data (*R* = 0.998) and exhibited 94% sensitivity and 88% specificity for identifying bacterial infections or coinfections. Gross hemolysis and icterus did not affect BV results, but gross lipemia showed considerable bias in samples with a moderate likelihood of viral etiology. BV outperformed WBC counts, PCT, and CRP in classifying bacterial infections.

A double-blind multicenter study was conducted using serum remnants collected from 5 pediatric emergency departments and 2 wards from children with (n = 529) and without (n = 68) suspicion of acute infection [31]. The 3 biomarkers used in BV distinguished between viral and bacterial infections with 93.8% sensitivity and 89.8% sensitivity, outperforming CRP and PCT. A study in *The Lancet* that same year [29] concluded that CRP, TRAIL, and IP-10 had the potential to reduce antibiotic misuse in young children based on a double-blind, international, multicenter study (n = 777, average age 21 months) with lower respiratory tract infections or fevers of unknown etiologies. A similar multicenter pediatric cohort study conducted in 2022 (n = 1140; mean age, 3.5 years) cited 93.7% sensitivity, 94.2% specificity, 73.0% PPV, and 98.9% NPV [24]. BV detected bacterial immune responses in viral polymerase chain reaction (PCR)–positive patients [32]. Among adults with suspected lower respiratory tract infections (N = 415, median age 56 years), BV exhibited 98.1% sensitivity, 88.4% specificity, and 98.8% NPV for bacterial infection [27].

## mRNA BIOMARKER EVALUATIONS

Gene expression is measured by quantifying mRNA, which, like the proteins translated from mRNA, can be used as biomarkers to characterize various infection types, since each pathogen activates a different set of host genes. Numerous studies have evaluated host transcriptomic approaches for the diagnosis of infectious diseases. Among studies evaluating transcriptomics for the diagnosis of pharyngitis, Tsalik and colleagues [33] identified 45 host mRNA transcripts that were found to be differentially expressed in viral, bacterial, or noninfectious causes of ARIs [33, 34]. A panel using multiplex reverse-transcription PCR (RT-PCR) to amplify these mRNAs of interest was developed and evaluated in 623 participants with bacterial, viral, coinfection, and noninfectious illness [35]. The RT-PCR test yielded an average weighted accuracy of 80.1% (bacterial infection) and 86.8% (viral infection), which was superior to PCT (68.7%; *P* < .001) [35]. The RT-PCR test has the potential to produce results at POC within 1 hour.

## LEUKOCYTE BIOMARKER EVALUATION

Recently, a rapid 4-color quantitative flow cytometric single-tube method was developed to detect bacterial infections within 30 minutes using host biomarkers (neutrophil complement receptors CD35, CD64, CD329) and major histocompatibility complex class I expression on blood leukocytes obtained from heparin blood samples [4]. Quantitative test results were used to calculate a 4-color bacterial infection (FCBI) index to confirm bacterial infections in 841 febrile patients with suspected infections. This novel method proved superior to CRP and PCT in distinguishing between microbiologically confirmed bacterial (n = 193) and viral (n = 291) infections. PCT was increased in 89% (95% confidence interval [CI], 83%–95%) of the bacteremic patients while CRP and the FCBI index detected bacteremia in the same cohort at 84% (95% CI, 77%–91%) and 94% (95% CI, 90%–98%), respectively. Additionally, bacterial and viral coinfections were quantifiable by FCBI index. Bacterial coinfection with virus was suggestive in 269 confirmed viral respiratory tract infections, with 43% of the patients exhibiting an increased FCBI index indicative of bacterial infection. Conversely, of a total of 193 patients with microbiologically confirmed bacterial infections, 11 also had concomitant confirmed viral infection. Ten of these 11 (91%) had an FCBI index ≥1.0, indicating that simultaneous viral infection did not have a significant influence on the detection of bacterial infection when using the FCBI index.

## EXOGENOUS NANOPARTICLES EVALUATION

Researchers at the Massachusetts Institute of Technology developed 20 nanoparticle sensors to distinguish between bacterial and viral pneumonia within 2 hours using a noninvasive urine sample [6]. By collecting 33 publicly available datasets of genes that are expressed during respiratory infections, they were able to identify 39 proteases that respond differently to viral and bacterial infections [36]. The nanoparticles were coated with peptides that are cleaved by the proteases of interest, causing the release of different reporter molecules. The reporter molecules are deposited into the lungs by intratracheal instillation, then excreted in the urine, which is analyzed via mass spectrometry. Using machine learning to analyze the data, they developed algorithms that could differentiate between individuals with pneumonia versus healthy controls and whether the infection was viral or bacterial. These proof-of-concept trials were conducted in mice where the nanosensors were injected directly into the trachea. To avoid invasive techniques such as injection or intratracheal instillation, Anahtar et al [6] are developing nebulizers for easier administration and lateral flow devices for a urine-based readout [36].

## BIOMARKER VALIDATIONS NEEDED

While host response biomarkers can be helpful in distinguishing bacterial versus viral infection and active infection from colonization, their role in GAS diagnosis and treatment remains undetermined. Many biomarkers (both novel approaches and those in clinical use) do not distinguish between infection and noninfection states. For infections, biomarkers such as CRP, PCT, and WBC are often used to assess the presence and severity of infection. However, these biomarkers can also be elevated in noninfectious conditions such as inflammation, trauma, or certain autoimmune disorders. This lack of specificity means that while these biomarkers can indicate an inflammatory response, they do not conclusively differentiate between infection and other causes of inflammation.

Because many bacterial and bacterial–viral infections are self-limiting, antibiotic stewardship only requires simple identification of the antibiotic-requiring infections rather than a complete description of the complex etiology of each infection [37]. With the development of more sensitive tests, there is concern that more antibiotics will be prescribed for self-limiting bacterial or viral–bacterial coinfections that would previously have gone undetected by culture or antigen methods [37]. According to a recent report, newer biomarkers have exhibited higher performance, but their lack of availability and higher price, as well as CLIA status, may make them inaccessible [7]. Therefore, future studies must identify the settings in which POC inflammatory response testing is most appropriate. Additionally, future studies evaluating the performance of novel biomarkers should have clearly defined criteria to determine infected versus noninfected status and evaluate impacts on laboratory and antimicrobial stewardship. Of critical importance, these studies must be performed in real-world scenarios (eg, where testing will actually be performed and using non-laboratory-trained personnel) to reliably assess test performance. Diverse groups from both sexes, patients with underlying conditions that mimic infection (eg, asthma), and immunosuppressed patients must also be included in future investigations of diagnostic methods based on gene expression [37]. To facilitate comparisons between clinical studies, a bacterial reference standard also needs to be adopted [37]. Future testing also needs to account for the impact that emerging pathogens, such as severe acute respiratory syndrome coronavirus 2, may have on the sensitivity and specificity of these approaches.

## FACTORS IMPACTING BIOMARKER PERFORMANCE

Several factors can significantly impact the performance and reliability of biomarkers used for detecting infections. These factors need careful consideration during the design, implementation, and interpretation of clinical studies involving biomarkers for infection. Factors in the preanalytical, analytical, and postanalytical phases of testing that can impact biomarker performance can include:

- Type of infection: Different infections and pathogens may induce varying immune responses and biomarker profiles. For example, bacterial infections like *S pyogenes* might elicit different biomarker responses compared to viral infections like rhinovirus. The specificity of the biomarker for a particular type of infection is crucial.
- Timing of testing: The stage of infection when the biomarker is measured can affect its performance. Biomarker levels may vary throughout the course of infection (eg, initial response, peak phase, resolution phase), influencing diagnostic accuracy.
- Patient characteristics: Patient demographics, previous exposures of the immune system, microbiome, geography, underlying health conditions (eg, immunocompromised status), and medication use (eg, antibiotics) can alter biomarker levels and their diagnostic performance.
- Clinical context and prevalence: The prevalence of the infection in the study population can impact the PPV and NPV of the biomarker. In low-prevalence settings, even a highly sensitive biomarker may yield false positives.
- Comorbidities and coinfections: Patients with comorbidities or coinfections may exhibit altered biomarker responses, complicating interpretation. For example, patients with chronic inflammatory diseases may have elevated baseline levels of inflammatory biomarkers.
- Biomarker assay variability: Variability in biomarker assay techniques, including sample collection methods (blood tubes or specimens collected via phlebotomy vs capillary), storage conditions, assay platforms, and calibration procedures, can introduce measurement variability and affect reproducibility.
- Biological variability: Inherent biological variability in biomarker levels among individuals can affect diagnostic accuracy. Biomarkers with high variability may require larger sample sizes to detect clinically meaningful differences.
- Interference factors: Nonspecific factors such as hemolysis, lipemia, icteria, medications, and other physiological conditions can interfere with biomarker measurements, leading to inaccurate results.
- Integration with clinical assessment: Biomarkers should complement clinical assessment rather than replace it entirely. Clinical judgment remains crucial in interpreting biomarker results in the context of patient history, physical examination findings, and other diagnostic tests.

## TACTICAL APPROACHES TO BIOMARKER EVALUATION AND DIAGNOSTICS

A novel biomarker test that could be performed on the throat swab sample would be ideal since the biomarker signal would represent cellular responses at the site of infection (eg, patients presenting with pharyngitis typically do not require venipuncture for laboratory diagnostics). Likewise, a site-of-infection swab may potentially limit carrier issues created by a more global test using blood or urine. Gene expression based on biofilms and quorum sensing may help distinguish between colonization/carrier and infectious cases. Tests that quantify, rather than simply detect, may prove more useful in predicting infections and treatment outcomes. Quantitation may allow clinicians to set ranges and cutoff levels that may reduce the effects of colonized carriers. In a tactical approach, POCTs for pathogen detection and host biomarkers could be used to create decision trees based on large population studies—algorithms that compare tests with patient vignettes, symptom scores, diagnoses, and outcomes.

Beyond specimen collection and test methodology, the location of testing is crucial to uptake of novel biomarkers for use in patients with pharyngitis. To optimize the patient experience, test results must be available during the time of visit and completed in ≤30 minutes (eg, a virtual visit is used and the patient presents to a pharmacy or laboratory for testing). Additionally, tests must be CLIA-waived in order to broaden the locations where POCT can be performed, thereby minimizing specimen transportation and testing times. For tests to be CLIA-waived, the test must use a nontechnical methodology (eg, be simple to perform), pose no harm to the patient even if it is performed incorrectly, and be cleared by the US FDA for “home use” (or other country-specific regulatory bodies). It must also include instructions that can be followed without deviation by any testing operator and reagents that are stable across a broad array of temperatures (room temperature storage is ideal), and provide clear and concise results that are readily interpretable.

## SUMMARY OF LABORATORY AND POC DIAGNOSTICS FOR PHARYNGITIS

In this 3-part supplement, the authors cite numerous pathogens and their respective occurrence frequency associated with pharyngitis. Although these primarily include commonly encountered bacterial and viral etiologies, emphasis was also given to those of emerging and reemerging potential due to declining vaccination rates and the speed at which they can be disseminated across the globe due to expanded regional and international travel. Traditional laboratory methods to detect these pathogens are relatively inexpensive but result in significant delays in diagnosis, pathogen-specific therapeutic intervention(s), and spread of disease throughout communities. The last 15 years has firmly established the widespread adoption of NAA diagnostics along with Centers for Medicare and Medicaid Services–approved reimbursement strategies. We are now entering the era of rapid/ultrarapid NAA diagnostics such that test results will be available within 5 to 30 minutes and in the POC setting. A key consideration is deciding which pathogens to include in these rapid NAA diagnostic testing solutions. Despite these technologic advancements, all currently available diagnostic testing solutions (rapid antigen, culture, NAA) cannot distinguish acute infection from colonization. This leads us into the rapidly emerging field of biomarkers. The clinical implementation of select biomarkers that can rapidly and accurately distinguish between colonization and infection due to bacterial and/or viral etiologies is a much sought-after diagnostic testing solution. In the future, diagnostic testing solutions for pharyngitis and other infectious diseases will likely utilize specific pathogen detection modalities in conjunction with biomarkers to reliably distinguish active infection from colonization. It is highly likely that this novel approach will be supported with AI solutions.

## Notes

***Author contributions.*** All authors contributed to this manuscript.

***Financial support*.** Educational funding for this article was provided by QuidelOrtho.

***Supplement sponsorship.*** This article appears as part of the supplement, “Laboratory and Point-of-Care Diagnostics for Pharyngitis: Pros and Cons?,” sponsored by QuidelOrtho.
